# A qualitative assessment of readiness to sustain Rapid Start ART in 14 publicly funded HIV clinics in the United States

**DOI:** 10.1186/s43058-026-00863-9

**Published:** 2026-01-15

**Authors:** Andres Maiorana, Alicia Bolton, Kimberly Koester, Beth Bourdeau, Lori DeLorenzo, Greg Rebchook, Wayne Steward, Susa Coffey, Oliver Bacon, Janet Myers

**Affiliations:** 1 Center for AIDS Prevention Studies, Division of Prevention Science, University of California, San Francisco, 16th Street, 3rd Floor, San Francisco, CA 94143 USA; 2 Organizational Ideas, 951 Coalwood Way, Blacksburg, VA 24060 USA; 3 Division of HIV, Infectious Diseases and Global Medicine at San Francisco General Hospital, University of California San Francisco, 350 Parnassus Ave, San Francisco, CA 94143 USA; 4 School of Medicine, HIV Division at San Francisco General Hospital, University of California San Francisco, 3333 California Street, Rm S111, UCSF Box 0874, San Francisco, CA 94118 USA

**Keywords:** HIV linkage intervention, Readiness to sustain Rapid Start ART, Sustainability strategies and factors, Sustainability framework

## Abstract

**Background:**

Current standards advise starting HIV antiretroviral therapy (ART) as soon as possible with the goal of achieving viral suppression. Applying the domains of a sustainability framework as a roadmap, we examine factors and strategies impacting readiness to sustain Rapid Start ART (RS-ART) across 14 sites participating in a national initiative that successfully implemented this intervention to link people with HIV to initiate ART treatment within seven days after linkage or re-engagement in care. While sustainability entails the ongoing delivery of a previously implemented intervention, factors and strategies for sustaining RS-ART have not been well-defined or studied.

**Methods:**

We conducted one-on-one semi-structured interviews with a purposeful sample of key informants from each of the 14 sites. Data were organized using Dedoose and analyzed using thematic analysis.

**Results:**

We conducted a total of 27 interviews with decision-makers and key staff implementing RS-ART. We identified a continuum across the sites, reflecting three different stages of readiness to sustain RS-ART. “Well-oiled machine” sites had comprehensive sustainability plans in place with RS-ART established as their current standard practice, supported by secured funding and organizational capacity. “On track” sites demonstrated a clear vision toward sustaining RS-ART, with progress contingent on securing funding and finalizing staffing plans. “To be determined” sites faced challenges, expressing uncertainty about obtaining necessary funding and determining sufficient human resources to sustain RS-ART. While feasibility and acceptability of RS-ART, driven by improved service and patient outcomes, were high across all sites, available funding and the necessary human resources were the two critical, interrelated factors impacting readiness to sustain RS-ART.

**Conclusions:**

Sites positioned to sustain RS-ART were able to secure funding for the necessary staff positions to effectively integrate it as standard of care. Future funding of HIV care programs must provide sufficient resources for all individuals to be offered RS-ART services to improve life expectancy, manage HIV as a chronic disease and prevent transmission to others. Given the dynamic nature of sustainability, future longitudinal studies are needed to evaluate RS-ART sustainability outcomes and its long-term effectiveness after it has been sustained.

**Supplementary Information:**

The online version contains supplementary material available at 10.1186/s43058-026-00863-9.

Contributions to the literature

- This article advances the underdeveloped area of sustainability research and evaluation within the field of Implementation Science
- This article examines evidence-based factors and strategies impacting readiness to sustain Rapid Start ART as a linkage intervention
- By applying the domains of a sustainability framework, this work provides a roadmap for organizations, translating complex implementation science concepts into clear, actionable strategies, for sustaining Rapid Start ART

## Background

Current standards for HIV treatment advise starting antiretroviral therapy (ART) as soon as possible following an HIV-positive test result [1, 2]. This strategy includes providing the necessary services to initiate ART shortly after linkage to, or reengagement, in care. Aptly called “Rapid Start”, this linkage intervention has an evidence base with numerous studies demonstrating its effectiveness in decreasing the time required to initiate treatment and to achieve viral suppression [3, 4]. Considering its proven benefits, this strategy is key to linking people with HIV to care and is a new standard of care for HIV services [5].

### The Rapid Start ART initiative

The “Building Capacity to Implement Rapid Start ART (RS-ART) for Improved Care Engagement in Ryan White HIV/AIDS Program” was a three-year (2020–2023) initiative funded by the Health Resources and Services Administration’s (HRSA) HIV/AIDS Bureau Ryan White HIV/AIDS Program Special Projects of National Significance (SPNS). The initiative has been described elsewhere [6]. Briefly, 14 sites in urban and rural settings throughout the US were funded to implement RS-ART. For the initiative, RS-ART was defined as linkage to care and initiation of ART within seven days for newly diagnosed, new to care and previously out of care persons with HIV (Additional file 1: Patient eligibility criteria). The initiative funded sites to implement RS-ART and provided support for existing or newly hired key staff, such as navigators or case managers, to facilitate linkage to care. The initiative also funded the University of California, San Francisco (UCSF) as an Evaluation and Technical Assistance Provider (ETAP) using an adapted model of the Institute for Healthcare Improvement’s Collaborative Model [7] to support RS-ART implementation. During implementation, sites sent service and patient outcomes to the ETAP (definitions of outcomes in Additional file 2), worked with an ETAP quality improvement coach and participated in twice or three times a year initiative-wide Learning Sessions (LS) and other technical assistance activities. Since there are no standard RS implementation guidelines [8], ETAP coaches supported sites in developing protocols tailored to their settings to ensure feasibility and consistent delivery. RS-ART sustainability was discussed in the two last LS in year three of the initiative. The coaches worked with the sites to develop sustainability action plans (according to the sites’ need to engage in that process) after the interviews, the primary data for this article, were conducted. Besides the coaches, an ETAP staff member served as liaison and point of contact with the sites. The liaisons participated, together with HRSA/SPNS project officers, in monthly monitoring calls and annual visits to assess implementation progress at the sites. All initiative activities were conducted online because of the Covid-19 pandemic.

### ETAP evaluation

Implementation Science methods guided the ETAP evaluation. The field of Implementation Science has emerged as “the scientific study of methods to promote the systematic uptake of research findings and evidence-based practices into routine practice,” [9] aiming to improve effectiveness in health services. The ETAP applied the Proctor’s Conceptual Model of Implementation Research [10] as a framework to study implementation outcomes among sites in the initiative. In this model, implementation outcomes (e.g. acceptability, feasibility, sustainability) result from applying strategies (actions and processes) that contribute to the successful implementation of a new intervention [11]. Implementation outcomes are indicators of implementation processes and preconditions impacting service and patient outcomes since an intervention may not be effective if not implemented well [10].

Sustainability is recognized as the end-goal of a successfully implemented intervention [12]. Sustainability entails the ongoing delivery of a previously implemented intervention, whose procedures and behaviors are either maintained or adapted, and results in benefits for patients and/or organizations [13]. The ongoing capacity to maintain or continue a service after initial funding ends is key to a definition of sustainability, as emphasized by different authors [14, 15].

Sustainability strategies consist of actions designed to influence the sustainment of health care interventions [16]. Compared to other implementation outcomes, sustainability outcomes and the strategies that may facilitate them have not been well-defined, conceptualized or studied empirically [17, 18]. Similarly, overlapping terms such as integration, institutionalization and sustainability may be confused or used interchangeably in the field of implementation science [17].

In this paper we examine the strategies and factors impacting readiness to sustain RS-ART in the sites participating in the HRSA/SPNS initiative. Since implementation science concepts and terms sometimes are complicated and abstract and may seem difficult to utilize in real life [19], we applied the domains of Schell and colleagues’ sustainability conceptual framework [14], developed through concept mapping and input from public health experts, to provide an easy-to-follow roadmap to our results in hopes that others find lessons for their own sustainability efforts. Because we conducted data collection associated with this analysis prior to the end of implementation of the initiative, we are limited to assessing readiness or the degree to which organizations were prepared to sustain RS-ART into longer-term programming.

## Methods

Qualitative research methods are well suited for understanding the factors that facilitate or inhibit sustainability [19, 20]. Our approach included semi-structured interviews with key informants as a primary data source to better understand the experiences and perceptions of those implementing and preparing to sustain RS-ART. We leveraged eight of the nine domains of the sustainability conceptual framework [14] to analyze the sites’ readiness and their capacity at an organizational level to sustain RS-ART. We selected this framework because of its interacting domains and ease of application. Its nine domains are: strategic planning, stable funding, organizational capacity, program adaptations, program evaluation, political support (buy-in), communication, partnerships and public health impact. We did not include the last domain (public health impact) because the sites were early in the process of sustainability when we conducted this assessment to evaluate impact over time after RS ART was sustained.

### Data collection

AM and AB, who also analyzed the data together with KK, conducted one-on-one semi-structured interviews with a purposeful sample of key informants from each site. We selected potential interviewees in consultation with the ETAP coaches, including decision makers and key implementing staff, to obtain a variety of perspectives based on their roles (medical providers, who in some cases were also the principal investigators for RS ART at their sites and project directors, as well as navigators and linkage coordinators). Informants were contacted by email. If they agreed to be interviewed, a virtual interview was scheduled. Everyone contacted agreed to be interviewed, but one person did not answer emails. Interviewees provided verbal consent before the interview and were offered a $50 gift card for their participation. The one-on-one interviews, lasting 45 to 60 min, were conducted and audio-recorded via Zoom between February/April 2023, and were transcribed verbatim by professional transcriptionists. The interviews were conducted using a semi-structured interview guide with questions tailored to the context of each site and informed by review of documents produced during the initiative (e.g. monitoring calls, site visit reports). The interview guide domains included reflections about implementation and factors impacting the sustainability of RS-ART services. The guide was informed by implementation science literature and broad sustainability concepts. While we did not explicitly identify Schell et al.’s sustainability framework domains (e.g., funding, organizational capacity) in the interviews, our questions mapped onto these areas, allowing us to capture participants’ perspectives without constraining participant’s answers into a technical framework. We report methods using the Consolidated Criteria for Reporting Qualitative Research Checklist [21] (Additional file 3). The study received Exempt Certification by the Human Research Protection Program at UCSF.

### Data analysis

We conducted a thematic analysis following an iterative process of reviewing transcripts, reading the literature and adjusting interpretations [22]. We leveraged eight of the nine domains of the sustainability conceptual framework [14] to analyze the sites’ readiness at an organizational level to sustain RS-ART. We selected this framework because of its interacting domains and ease of application. Its nine domains are: strategic planning, stable funding, organizational capacity, program adaptations, program evaluation, political support (buy-in), communication, partnerships and public health impact. We did not include the last domain (public health impact) because the sites were early in the process of sustainability when we conducted this assessment.

AM, AB and KK, anthropologists experienced conducting HIV evaluation research in medical settings, analyzed the data. They were familiar with the sites because of their role as site liaisons and/or having observed the periodic LS, which facilitated analysis and data immersion. The analysts first read a cross-section of the interview transcripts and developed a preliminary codebook with deductive and inductive codes respectively based on the interview guide and themes emerging from the data that they applied to a sub-set of interviews, modifying the codes during the process to reflect nuances in conceptual categories. They then agreed on a final set of codes that they applied independently to every interview transcript using Dedoose, an online data management program [23], periodically meeting to compare coding and resolve discrepancies by consensus. Interview field notes also informed the analysis.

Once coding was complete, the analysts summarized excerpts from a subset of codes (including implementation and sustainability as main codes and barriers, facilitators and adaptations as subcodes) to identify salient themes related to implementation and sustainability. We verified our findings by reviewing the sections of the last site visit reports pertaining to sustainability conducted towards the end of the initiative. Two of the five interviewees who were project staff could not provide specific information about future funding, staffing, etc. needed to sustain RS-ART. When perspectives differed across staff roles, we interpreted these differences as reflecting varying levels of knowledge, and we prioritized responses from project directors or principal investigators who were familiar with sustainability planning. Data informing readiness to sustain RS-ART were considered as prospective. Findings were reviewed with the sites during a grantees’ meeting at the end of the initiative as a “member check,” a strategy to validate qualitative findings [24].

## Results

We present data from 27 interviews with key informants implementing or supervising RS-ART implementation at the 14 sites participating in the HRSA/SPNS initiative. We conducted two interviews at 13 sites and one interview at one site based on the number of informants available (Table 1 below shows key informants’ characteristics).

**Table 1:** Key Informant Roles and Demographics (N = 27)

*Role in Rapid Start*	*N*	*Percent*
Project Director/Coordinator	17	(63%)
Project Staff (navigators, linkage coordinators)	4	(15%)
Data Staff	1	(4%)
Medical Provider/Principal Investigator	5	(19%)
***Sex***		
Male	12	(44%)
Female	15	(56%)
***Race/Ethnicity***		
Asian	2	(7%)
Black or African American	7	(26%)
Latinx or Hispanic	5	(19%)
White	12	(44%)
Latinx or Hispanic and White	1	(4%)
Total:	27	

(Table 2 below shows the type of organization by site).

**Table 2:** Number of sites and type of organization in the continuum of readiness to sustain RS-ART

Type	Well-Oiled Machine (WOM) Sites	On Track (OT) Sites	To Be Determined (TBD) Sites
Hospital (with outpatient clinic)	3	1	-
Federally Qualified Health Center	2	2	2
Community Based Organization (CBO)	1	1	2

We first present some implementation insights related to sustainability of RS-ART, to then, examining the sites’ readiness to sustain RS-ART categorized according to the domains of the sustainability conceptual framework.^23^

Key informants across all sites reported that RS-ART had become embedded into their services because it “had grown legs,” all staff knew what to do and it was no longer viewed as a special project. Their reasoning can be viewed as evidence of high levels of feasibility and acceptability of RS-ART, underscored by the improved service and patient outcomes resulting from its successful implementation [25]. Informants reported that key strategies for RS-ART implementation included the development of mechanisms for identifying patients for RS-ART; development of an RS-ART protocol; integration of RS-ART workflows into linkage, medical, laboratory and pharmacy procedures, including availability of ART starter packs for patients; hiring or leveraging appropriate implementation staff and ensuring buy-in and effective communication among staff. The availability of supportive services and paying sources for RS-ART services also were drivers of successful implementation. Some sites with more than one clinic already had expanded RS-ART services to several or all their medical facilities during the initiative, indicating penetration of the service into their organization as an implementation outcome [10].

While at the time of the interviews informants at all sites reported that RS-ART had been embedded into their routine services, not all sites appeared ready to sustain it. Based on these findings, we identified a continuum consisting of three different stages of readiness to sustain RS-ART. Informants at six (A-F) sites that we refer to as in the “Well-oiled machine” (WOM) stage, applying the term used by an interviewee referring to sustaining RS-ART at her site, outlined a specific, solid sustainability plan and considered RS-ART as current standard practice with funding sources and organizational capacity determined. With a vision in place, informants at the five (G-L) sites we refer to as in the “On track” (OT) stage, reported they were on their way to sustain RS-ART but had not yet secured funding and finalized staffing plans. At the remaining three sites (M–O) we subsequently refer as “To be determined” (TBD), interviewees raised concerns or were somewhat uncertain about next steps to procure funding and the human resources needed towards RS-ART sustainability.

Building on the continuum above, we examine the sites’ readiness to sustain RS-ART according to eight intersecting domains of the sustainability conceptual framework [14]. We present the eight domains in order of relevance to the factors and strategies related to sustainability in our data. We include implementation strategies in the last three domains also facilitating sustainability (Table 3 shows the sites’ continuum of readiness to sustain RS-ART).

**Table 3:** Sites’ continuum of readiness to sustain RS-ART following the domains of the sustainability conceptual framework

Sustainability Framework Domains	Well-Oiled Machine Sites	On Track Sites	To Be Determined Sites
Strategic Planning	Completed	In progress	In progress
Stable Funding	Secured	Pending or identified but not secured	Uncertain
Organizational Capacity	In place	Pending (Contingent on funding)	Uncertain, with not defined plans
Evaluation	On-going	Pending (Contingent on funding)	Uncertain, with not defined plans
Adaptations	No change to RS-ART core premise (treatment within seven days). Other modifications related to staffing and procedures at some sites		
Political Support	Internal support and external support (funding) in place	Internal support in place	Internal support in place
Communication	In place from implementation	In place from implementation	In place from implementation
Partnerships	In place from implementation	In place from implementation	In place from implementation

1. Strategic Planning

This domain encompasses setting goals and formulating strategies to ensure sustainability of an intervention. In this case, all sites had or were engaged in a process of planning to sustain RS-ART after completion of the initiative influenced by positive service and patient outcomes resulting from RS-ART implementation. These processes, akin to delineating program direction, varied according to the sites’ organizational structures. Planning involved developing a financial strategy, identifying funding sources and determining staffing. The project director at WOM Site A described efforts to secure funding well before the end of the initiative, emphasizing the importance of program data to obtain stakeholders’ support to sustain RS-ART in concordance with the agency’s mission to end the HIV epidemic:

When we started to see the outcomes on our quality measures: seeing that return-to-care patients weren’t waiting 48 days to start medication, it was [instead] like four days. I was able to take that to our leadership team, our client advisory board, the implementation team, the clinical and the grants and finance teams. When I was able to show them the patient outcomes, it became like, ‘Wow, why have we not been doing this?’ It became a standard of care as to how we care for patients.

2. Stable Funding

As key findings from our data in this domain and organizational capacity below, available funding and the necessary human resources were the two interrelated main factors reported by our interviewees influencing readiness to sustain RS-ART. To sustain it, the sites had to transition to stable funding sources to cover staff and other expenses. WOM sites had already secured alternative funding sources. Their financial strategies included seeking funding from existing HRSA Ryan White Programs such as Part A, Part C and the 340B Drug Pricing Program, grants available through “Ending the HIV Epidemic” initiative, local health departments and insurance networks’ revenue. While affected by drug pricing and regulatory changes, the 340B Program provides flexible and discretionary funding to medical settings as covered entities [26]. Some sites used a combination of funding strategies, including fundraising or dividing costs across funding streams or departments within the organization. The medical director at WOM Site B planned to use samples of ART available from pharmaceutical companies to ensure a reliable supply of startup ART packets for patients.

OT and TBD sites were in the process of either securing or trying to identify funding. OT sites had identified or were in the process of identifying funding sources (in some cases similar to the ones secured by the WOM sites) but were waiting to secure that funding to finalize staffing plans. TBD sites, on the other hand, had not yet identified funding and were uncertain about staffing plans. The project director at OT Site G, while trying to secure funding to support key staff, prioritized sustaining RS-ART as part of their services:

Sustaining Rapid Start itself [medical and non-medical workflows and procedures] is not the difficult part. Providers are used to the protocol. They know to do the [ART] startup packs. It’s kind of second nature to them. Rapid Start is the doorway to all the services [we] provide…. If this [RS-ART] can’t continue, we might as well just shut the place down. The sustainability piece we are working on is how to pay our nurse and care coordinator: key positions that we need: the captains of this ship.

And the project director at TBD Site M, not yet having identified funding explained:

The only thing that hinders it [RS-ART] is financially. What funds are we going to use? That’s what’s in the talks. Looking at grants. Where can we add staff? Or keep staff. We’re going to try very hard. That’s our priority.

3. Organizational capacity

Defined as the resources necessary for effectively managing program activities, organizational capacity, mainly staffing, played a pivotal role in sustaining RS-ART. Available funding would determine the human resource strategies to sustain RS-ART. Strategies at some WOM sites included moving the dedicated implementation staff funded by the initiative into permanent positions written into their budgets, although with potential changes in roles or scope of work. The medical director at WOM Site B explained that they had planned for sustainability in advance: “It's completely sustainable. The personnel that work at Rapid is permanent now. Most of the stuff we needed is personnel infrastructure. It's like we wrote it. They would share responsibilities with other projects so that they can be sustained.”

A couple of WOM sites planned to only keep a case manager, navigator or outreach worker as “critical” staff needed to ensure linkage to care, with supervisory and data monitoring roles covered by staff not previously funded by the initiative. At WOM Site C, a large academic organization, a Medical Intake Coordinator position created during RS-ART implementation to centralize scheduling and monitoring appointments was set to become permanent.

Other WOM sites cross-trained staff to continue implementation and prevent RS-ART service interruptions. The project director at WOM Site A, emphasized the importance of creating a culture in which staff members acquire transferable skills to provide different services as required in case of staff turnover or public health emergencies. The program manager at WOM Site D, a status-neutral clinic providing prevention services and care in the same setting independent of HIV status, explained:

Everyone is on board and wants this [RS-ART] to remain. To have this status-neutral clinic is a big deal. Now all our staff are able to do it [RS-ART], new staff gets trained in both pre-exposure prophylaxis (PrEP) and test and treat. It’s part of our protocols. It’s part of our workflow.

OT and TBD sites, without having secured stable funding, either had not finalized or defined staffing strategies. Some voiced concerns about the impact of staffing changes on RS-ART services due to losing initiative funding, including not retaining key RS-ART staff and the risk of overwhelming a single remaining staff member, such as a navigator, with an excessive workload and patient volume. The HIV services director at OT Site G noted that reallocating their two RS-ART full time staff (nurse manager and care coordinator) to other duties may lead to RS-ART services being deprioritized. The project director at OT Site H stated the need to secure funding for their existing staff and having an alternative sustainability plan:

We used existing staff to implement Rapid, so we don’t see why the institution would not continue to fund that staff member. We kind of have a plan for the existing staff. Leadership plans to probably apply for more funding to maintain the program manager and a data person. I do think that 95 percent – It’s going to happen. But we need a contingency [plan] if we don’t receive additional funding.

4. Program evaluation

Evaluation, a required and critical component during the initiative, encompassed the monitoring of process and outcome data associated with RS-ART services. Informants at most sites stated that their organizations planned or intended to continue to collect data as part of sustaining RS-ART to evaluate its ongoing impact. Utilizing some of the structures and measures developed by the ETAP for the initiative would facilitate those evaluation efforts. OT and TBD sites, nevertheless, because of their unfinished planning and funding, had not decided whether they would continue monitoring or what data to include in that monitoring.

5. Adaptations

This domain refers to the necessary adaptations to ensure intervention effectiveness. All sites planned to continue RS-ART without changes to the core premise: initiating ART within seven days of diagnosis or engagement in care. Adaptations to sustain RS-ART depended on the sites’ particular characteristics, including staffing adjustments at the end of the initiative. A couple of sites shortened the RS-ART initial medical visit to focus on starting ART, with other medical exams and services covered in subsequent appointments. At some sites, telemedicine for initial medical visits, as instituted during the Covid-19 pandemic, remained an option. At least one site planned to expand the RS-ART criteria to include some individuals previously out of care, not eligible under the initiative’s criteria.

6. Political support

This domain is defined as the internal support, buy-in and acceptability and the external support of an intervention. During RS-ART implementation, sites had secured organizational support from leadership, fostering overall buy-in and ensuring clinicians’ comfort in prescribing RS-ART. Internal champions were instrumental to this process, as their leadership and/or commitment were essential in securing support for RS. With buy-in already in place from implementation, the sites did not develop specific sustainability strategies related to internal support in this domain. Internal support, influenced by positive service and patient outcomes, resulted, as the project director at WOM site E stated, in RS becoming policy and standard practice at WOM sites.

Now [RS-ART] it’s part of our day-to-day operations. It’s a component of the services we offer. The outcomes of the patients who begin rapid initiation have been immensely improved getting them into care. When it becomes policy within the organization…. It has become a standard form of practice here.

At TBD Site M, however, an organization with multiple clinics covering a large geographical area, the director of medical services acknowledged that while buy-in and having an RS-ART protocol were key, navigating internal politics and integrating RS-ART into organizational policies may take time, and depend on champions like himself promoting it within the organization.

Having positive service and outcome data, besides contributing to internal support, may facilitate securing external support. The intake coordinator at WOM Site F stated: “One thing that is cool is we do have the data. We do have the story. We have something to sell as far as getting funding.” While still early in the process of seeking funding, the executive director at TBD Site N, explained that potential changes to policies and rebates related to 340B funds may affect external support and receiving funding to pay staff and sustain RS-ART.

7. Communication

Effective communication among stakeholders about RS-ART implementation and dissemination was important in all the sites. Implementation strategies such as developing an RS-ART protocol enhanced internal communications. With processes in place, the medical director at site WOM B considered the protocol developed during the initiative as a live document perhaps to be revisited in the future. For her, the utilization of the protocol would also contribute to sustaining RS-ART by facilitating training and onboarding of medical and non-medical staff on written standard procedures. Large sites promoted RS-ART internally, in a few cases through in-house videos to facilitate referrals within the organization. RS-ART champions, particularly medical providers, played an important role promoting and disseminating RS-ART internally and externally through formal and informal communication channels with other medical providers and medical residents. Several sites used health information technology as a key strategy to enhance systematic internal communication, utilizing panel management and including RS-ART referrals and tracking built into their electronic medical records (EMR). For example, OT Site I, a large setting, designed a streamlined process using alerts and a six-question survey embedded in their (EMR) to facilitate RS-ART referrals at their six medical facilities.

Some sites marketed RS-ART branding and creating and identity for the service on their websites. Many sites engaged their Community Advisory Boards (CAB) to promote RS-ART in the community. One site had sought input from their CAB, which included clients, on their Patient Welcome Packet, containing information on RS-ART and aiming to facilitate referrals through community partners. Another site planned to sustain the involvement of peer mentors to provide the lived experience support for patients receiving RS-ART services and to promote it in the community. The sites did not develop specific sustainability strategies in this domain or the one above, but strategies to develop internal support and effective communication intertwined to contribute to successful implementation and to sustain RS-ART.

8. Partnerships

This domain includes external collaborations and connection with community. While RS-ART is a clinic-based intervention, throughout implementation sites leveraged their relationships with HIV testing sites, community agencies and local health jurisdictions to extend outreach and coordinate incoming referrals for RS-ART as well as outgoing referrals to other services to cover patient needs. Developing partnerships intrinsically related to internal political support and communication (the previous two domains). Partnerships were key at Community-Based Organization (CBO) sites that previously did not provide HIV care. One OT CBO started offering short-term HIV care while implementing RS-ART within the context of their PrEP services and expanded its partnerships to enhance outreach, boost incoming referrals and ensure outgoing referrals for long-term HIV care. The project director at a WOM CBO spoke of their partnership to provide bridge care, identifying individuals for RS-ART at the CBO who received care at their partner clinic, as a facilitator of RS-ART sustainability:

Having [CBO] staff seated at [partner clinic] has just been a huge resource and is going to help us increase our sustainability. During Rapid, we changed our hours. We are open late a couple of days a week. The pharmacy is open later. It’s [CBO] and [partner clinic] and the pharmacy working together for the common goal of meeting the needs of patients.

## Discussion

Our findings reflect a continuum of readiness to sustain RS-ART across the 14 sites participating in the HRSA/SPNS initiative. With the necessary planning, readiness was mainly associated with available funding and organizational capacity. “Well-oiled machine” sites had secured funding and RS-ART already had become standard practice. The main difference between readiness to sustain RS-ART at OT and TBD sites related to funding. OT sites showed a clear vision and defined staffing plans towards sustaining RS-ART; they already had identified or were in the process of identifying funding sources but not quite yet secured that funding, while TBD sites had not identified funding and next steps to procure funding and define staffing plans were uncertain.

Applying interrelated domains from a sustainability framework, we present strategies and factors related to the process of readiness to sustain RS-ART. Specific sustainability strategies and factors pertained to the first four domains (strategic planning, stable funding, organizational capacity and data monitoring). Most sites, benefitting from the evaluation resources created by the initiative, intended to sustain data monitoring in the fourth domain to evaluate RS-ART programming over time. Regarding adaptations in the fifth domain, the sites did not plan to adapt the premise of RS-ART to start or restart ART within seven days. Reflecting a continuity between feasibility, acceptability and sustainability, successful strategies in place developed during implementation pertaining to the remaining domains (political support, communication and partnerships). contributed to readiness to sustain, transforming into de facto sustainability strategies.

Our findings align with the literature on intervention sustainability [27] whereby funding and staffing are highly interrelated factors that either facilitated readiness to sustain at WOM sites or hindered it at OT and TBD sites. This suggests that stable funding and a workable staffing structure as organizational capacity may be determinants or predictors of RS-ART sustainability. Also as interacting domains, strategic planning, stable funding and organizational capacity interweave with internal support and the will to sustain RS-ART. WOM sites positioned to sustain RS-ART shared key similarities, including proactive planning and the development of strategies to secure stable funding and maintain organizational capacity through appropriate staffing. Organizations with access to multiple funding sources (e.g., 340B Program and revenue from different funding streams and insurance networks) and well-developed staffing plans, including cross-training personnel to deliver various services, are better positioned to sustain an RS-ART program. Conversely, organizations without robust funding sources, with limited personnel or experiencing staff turnover may struggle to effectively sustain RS-ART.

The sites’ readiness to sustain RS-ART reflect the overlapping institutionalization stages of a program [28] as reflected by: 1) plans for transitioning from HRSA SPNS funding to other financial sources, mainly for staff; 2) RS-ART becoming a routine and standardized set of services for ART initiation; and 3) integration of RS-ART into the organizations’ policy and culture as a regular practice, including developing a protocol to preserve institutional knowledge. Feasibility and acceptability, with workflows, procedures and internal support in place, as positive implementation outcomes were measures of success [12] that impacted the sites’ decision to sustain RS-ART. Demonstrating positive service and patient outcomes, together with the commitment to integrate RS-ART as standard of care based on current guidelines, may improve the outlook for securing external support and funding.

This study presents several limitations. Our results may not be transferrable to organizations without the infrastructure and support provided by the HRSA/SPNS initiative; however, they provide a roadmap and illustrate key issues to consider for sustaining RS-ART. While our interview guide asked broad questions about sustainability, applying the domains of a sustainability framework to our analysis provided a structure to examine readiness to sustain RS-ART. The sustainability strategies were more defined at the WOM sites, and less distinct at OT and TBD sites. We did not collect details about sites’ strategic planning process, stakeholders involved in that planning or other organizational issues that may have influenced sustainability readiness. Similarly, the specifics of the external support received by WOM sites or the sources of financial support sought by OT and TBD sites are less defined in our data. Also because the sites were early in the process of sustainability, we could not determine the ongoing or long-term impact of RS-ART as an intervention to link people to HIV care and prompt access to ART as a sustainability outcome. However, the sites had achieved or were in the process of achieving other sustainability outcomes (some of which overlap with the sustainability domains we analyzed and summarized in Table 3) identified in a review of sustainability approaches [29], including stable funding, organizational capacity and scaling up of RS-ART.

Sustainability is an iterative and dynamic process in an always changing health care environment [18] and the nature of available funding may bring uncertainty to staffing and other aspects of health care delivery. Sustainability cannot be completely assessed using data from implementation studies that stop at the end of a grant like ours [12]. Sustainability strategies and outcomes should be evaluated longitudinally [30] to understand the longer term impact of an intervention such as RS-ART.

## Conclusions

HIV treatment guidelines have dramatically evolved over time as understanding of HIV pathogenesis and management of HIV disease have progressed and new pharmaceutical regimens and delivery methods become available. The offer of HIV treatment at the time of diagnosis has become standard of care in the U.S but gaps in implementation remain. The HRSA/SPNS initiative has given clinics serving the most vulnerable patients a chance to implement RS-ART services, building capacity and facilitating practice transformation to sustain RS-ART going forward. Other clinics can benefit from the key lesson learned: with planning, internal support, procedures in place and improved outcomes, sustainability depends on secure funding for the necessary staff positions to effectively integrate RS-ART as standard of care. Considering this, models for funding HIV care should provide sufficient resources to ensure the capacity to provide RS-ART because it delivers on the promise of access for all. Individuals, regardless of whether they are seeking care in an academic medical center, a community-based clinic or a rural health center, can benefit from RS-ART services to improve their health, avoid transmission to others and contribute to the end of HIV/AIDS.

## Supplementary Information

Additional file 1: Patient eligibility criteria, docx.

Additional file 2: Service and outcome definitions, docx.

Additional file 3: COREQ list, docx.

Additional file 4: Interview guide.

## Data Availability

The data collected and analyzed for this study are not publicly available to protect participant confidentiality.

## Abbreviations

ART: Antiretroviral therapy
RS-ART: Rapid Start ART
HRSA: Health Resources and Services Administration
SPNS: Special Projects of National Significance
UCSF: University of California San Francisco
ETAP: Evaluation and Technical Assistance Provider
LS: Learning Sessions
AM: Andres Maiorana
AB: Alicia Bolton
KK: Kimberly Koester
WOM: Well Oiled Machine
OT: On Track
TBD: To Be Determined
PrEP: Pre-exposure prophylaxis
EMR: Electronic Medical Records
CAB: Community Advisory Boards
CBO: Community-Based Organization

## References

[1] Panel on Antiretroviral Guidelines for Adults and Adolescents. Guidelines for the Use of Antiretroviral Agents in Adults and Adolescents with HIV.: Department of Health and Human Services.; 2024 [Available from: https://clinicalinfo.hiv.gov/en/guidelines/adult-and-adolescent-arv.

[2] Organization WH. Guidelines for managing advanced HIV disease and rapid initiation of antiretroviral therapy, July 2017. Geneva; 2017. 29341560

[3] Doshi RK, Greenberg AE. Test, treat, and maintain: rapid initiation of antiretroviral therapy. AIDS (London). 2021;35(11):1867–9. 10.1097/QAD.0000000000002994 PMC8459936 34397486

[4] Halperin J, Butler I, Conner K, Myers L, Holm P, Bartram L, et al. Linkage and antiretroviral therapy within 72 hours at a federally qualified health center in New Orleans. AIDS Patient Care STDs. 2018;32(2):39–41. 29432044 10.1089/apc.2017.0309 PMC5808385

[5] Coffey S, Halperin J, Rana A, Colasanti J. Rapid antiretroviral therapy: time for a new standard of care. Clin Infect Dis. 2021;73(1):134–6. 32777033 10.1093/cid/ciaa1171

[6] Bourdeau B, Shade SB, Koester KA, Rebchook GM, Steward WT, Agins BM, et al. Rapid start antiretroviral therapies for improved engagement in HIV care: implementation science evaluation protocol. BMC Health Serv Res. 2023;23(1):503. 37198586 10.1186/s12913-023-09500-w PMC10189984

[7] The Breakthrough Series. IHI’s Collaborative Model for Achieving Breakthrough Improvement. Boston: Institute for Healthcare Improvement; 2003.

[8] Michienzi SM, Barrios M, Badowski ME. Evidence regarding rapid initiation of antiretroviral therapy in patients living with HIV. Curr Infect Dis Rep. 2021;23(5):7. 33824625 10.1007/s11908-021-00750-5 PMC8016613

[9] Eccles MP, Mittman BS. Welcome to Implementation Science. Implement Sci. 2006;1(1):1.

[10] Proctor E, Silmere H, Raghavan R, Hovmand P, Aarons G, Bunger A, et al. Outcomes for Implementation Research: Conceptual Distinctions, Measurement Challenges, and Research Agenda. Administration and policy in mental health and mental health services research. 2011;38(2):65–76. 20957426 10.1007/s10488-010-0319-7 PMC3068522

[11] Proctor E, Powell B, McMillen J. Implementation strategies: recommendations for specifying and reporting. Implement Sci. 2013;8:139. 24289295 10.1186/1748-5908-8-139 PMC3882890

[12] Proctor E, Luke D, Calhoun A, McMillen C, Brownson R, McCrary S, et al. Sustainability of evidence-based healthcare: research agenda, methodological advances, and infrastructure support. Implement Sci. 2015;10(1):88. 26062907 10.1186/s13012-015-0274-5 PMC4494699

[13] Moore JE, Mascarenhas A, Bain J, Straus SE. Developing a comprehensive definition of sustainability. Implement Sci. 2017;12(1):110. 28865479 10.1186/s13012-017-0637-1 PMC5581411

[14] Schell SF, Luke DA, Schooley MW, Elliott MB, Herbers SH, Mueller NB, et al. Public health program capacity for sustainability: a new framework. Implement Sci. 2013;8(1):15. 23375082 10.1186/1748-5908-8-15 PMC3599102

[15] Walugembe DR, Sibbald S, Le Ber MJ, Kothari A. Sustainability of public health interventions: where are the gaps? Health Res Policy Syst. 2019;17(1):8. 30646911 10.1186/s12961-018-0405-y PMC6334403

[16] Hailemariam M, Bustos T, Montgomery B, Barajas R, Evans LB, Drahota A. Evidence-based intervention sustainability strategies: a systematic review. Implement Sci. 2019;14(1):57. 31171004 10.1186/s13012-019-0910-6 PMC6554955

[17] Birken SA, Haines ER, Hwang S, Chambers DA, Bunger AC, Nilsen P. Advancing understanding and identifying strategies for sustaining evidence-based practices: a review of reviews. Implement Sci. 2020;15(1):88. 33036653 10.1186/s13012-020-01040-9 PMC7545853

[18] Shelton RC, Cooper BR, Stirman SW. The Sustainability of Evidence-Based Interventions and Practices in Public Health and Health Care. 2018(1545–2093 (Electronic)). 10.1146/annurev-publhealth-040617-014731 29328872

[19] Moullin JC, Sklar M, Green A, Dickson KS, Stadnick NA, Reeder K, et al. Advancing the pragmatic measurement of sustainment: a narrative review of measures. Implement Sci Commun. 2020;1(1):76. 32964208 10.1186/s43058-020-00068-8 PMC7499830

[20] Hamilton AB, Finley EP. Qualitative methods in implementation research: An introduction. 2019(1872–7123 (Electronic)). 10.1016/j.psychres.2019.112516 PMC7023962 31437661

[21] Tong A, Sainsbury P, Craig J. Consolidated criteria for reporting qualitative research (COREQ): a 32-item checklist for interviews and focus groups. Int J Qual Health Care. 2007;19(6):349–57. 17872937 10.1093/intqhc/mzm042

[22] Huberman AM, Miles MB. Qualitative data analysis: an expanded sourcebook. Thousand Oaks, CA: Sage; 1994.

[23] Dedoose Version 7.0.23. web application for managing, analyzing, and presenting qualitative and mixed method research data. Los Angeles, CA: SocioCultural Research Consultants, LLC; 2016.

[24] Lincoln Y, Guba EG. Naturalistic inquiry. Int J Intercult Relat. 1985. 10.1016/0147-1767(85)90062-8. Newbury Park, CA: Sage.

[25] Bolton A, Palomares M, Laporte-Sanchez X, Tobe C, Perkins J. Key Adaptations Made by Clinics Successfully Implementing a Rapid Start Approach to Accelerate ART Initiation. National Ryan White Conference on HIV Care & Treatment; Washington DC2024.

[26] Knox RP, Wang J, Feldman WB, Kesselheim AS, Sarpatwari A. Outcomes of the 340B Drug Pricing Program: A Scoping Review. JAMA Health Forum. 2023;4(11):e233716-e. 10.1001/jamahealthforum.2023.3716 PMC10665972 37991784

[27] Scheirer MA, Dearing JW. An agenda for research on the sustainability of public health programs. 2011(1541-0048 (Electronic)). 10.2105/AJPH.2011.300193 PMC3222409 21940916

[28] Rabin BA, Brownson RC, Haire-Joshu D, Kreuter MW, Weaver NL. A glossary for dissemination and implementation research in health. 2008(1550-5022 (Electronic)). 10.1097/01.PHH.0000311888.06252.bb 18287916

[29] Flynn R, Stevens B, Bains A, Kennedy M, Scott SD. Identifying existing approaches used to evaluate the sustainability of evidence-based interventions in healthcare: an integrative review. Syst Rev. 2022;11(1):221. 36243760 10.1186/s13643-022-02093-1 PMC9569065

[30] Alexander JA, Hearld LR. Methods and metrics challenges of delivery-system research. Implement Sci. 2012;7(1):15. 22409885 10.1186/1748-5908-7-15 PMC3317852

