# Supplementary material for: A qualitative assessment of readiness to sustain Rapid Start ART in 14 publicly funded HIV clinics in the United States
Source: Implement Sci Commun. 2026 Jan 15;7:29. doi: 10.1186/s43058-026-00863-9 (PMC12892499; doi:10.1186/s43058-026-00863-9)
Supplement: Supplementary file 1 — Additional file 1: Patient eligibility criteria, docx. [file 43058_2026_863_MOESM1_ESM.docx]

**Patient Eligibility Criteria for the “Building Capacity to Implement Rapid ART Start for Improved Care Engagement in Ryan White HIV/AIDS Program” initiative**

| **Patient population** | **Eligibility criteria** |
| --- | --- |
| **Newly Diagnosed​:** | Any person with a new positive HIV rapid, confirmatory test result within 12 months and not previously on HIV antiretroviral therapy. ​ |
| **New to care**: | Any person diagnosed with HIV greater than 12 months prior who has not previously attended a HIV care medical visit or has never been on ART. |
| **Returning to Care:** ​ | Any person diagnosed with HIV with previous engagement in HIV care not on HIV antiretroviral therapy for greater than 12 months, has no medical visit or laboratory test result for greater than 12 months​. |
