# Supplementary material for: A qualitative assessment of readiness to sustain Rapid Start ART in 14 publicly funded HIV clinics in the United States
Source: Implement Sci Commun. 2026 Jan 15;7:29. doi: 10.1186/s43058-026-00863-9 (PMC12892499; doi:10.1186/s43058-026-00863-9)
Supplement: Supplementary file 2 — Additional file 2: Service and outcome definitions, docx. [file 43058_2026_863_MOESM2_ESM.docx]

**Service and Patient Outcome Definitions for the “Building Capacity to Implement Rapid ART Start for Improved Care Engagement in Ryan White HIV/AIDS Program” initiative**

**Service outcomes:**

- **Initiation of ART in 7 days:** Percentage of patients with HIV newly diagnosed, new to care, and/or out of care who are prescribed HIV antiretroviral therapy within seven days from date of identification
- **Linkage to care in 7 days:** Percentage of patients with HIV newly diagnosed, new to care, and/or out of care who attended their first medical visit within seven days from date of identification
- **Retention to care:** Percentage of patients with HIV newly diagnosed, new to care, and/or out of care who initiated on ART with at least 1 medical visit in each six-month period at least 90 days apart

**Patient outcomes:**

- **Viral suppression:** Percentage of patients with HIV newly diagnosed, new to care, and/or out of care with a HIV viral load less than 200 copies/ml at last viral load test at a specified time after initiation of ART (either 60 days or 12 months)
- **Time to first viral suppression:** Number of days from ART start to the first viral load of less than 200 copies/ml
