# Supplementary material for: A qualitative assessment of readiness to sustain Rapid Start ART in 14 publicly funded HIV clinics in the United States
Source: Implement Sci Commun. 2026 Jan 15;7:29. doi: 10.1186/s43058-026-00863-9 (PMC12892499; doi:10.1186/s43058-026-00863-9)
Supplement: Supplementary file 4 — Additional file 4: Interview guide. [file 43058_2026_863_MOESM4_ESM.docx]

**Sustaining Rapid Start ART Key Informant Interviews**

Thanks for taking part in this interview! We are nearing the end of the HRSA/SPNS initiative and want to hear about how things are going with Rapid Start ART these days and we will focus in on sustainability of Rapid Start ART services at your organization. We are recording this interview, but your answers will remain confidential and your name will never be connected to your answers. Do you have any questions?

1. **Introduction**

- Just for the record, what is your job title and role within [organization name]?
- What is your current role in/involvement with the Rapid Start ART intervention?
  - How has your role in/involvement with Rapid Start ART evolved over the course of this initiative?
- How long have you been working at [organization name]?

1. **Current State of Rapid Start ART**

- Can you tell me about how things are going with Rapid Start ART implementation now?

*PROBES*

- Who is most closely involved in project operation now?
  - How has that changed over the life of the initiative?

*If team has changed, and (some) members are no longer active…*

- - What led to changes in team composition?
- Are you fully staffed?
- What does program operation look like right now, on a day-to-day basis?
- How are your workflows working out?
- At this point in time, what is the degree of buy-in of Rapid Start ART by people involved in it/at your organization?

1. **Reflections on Implementation Process/Experiences**

*Now that you have the benefit of some hindsight on this project, I’d like to ask you to reflect on the implementation process.*

- What came as the biggest surprise to you as you worked on rolling out/implementing Rapid Start ART (or just what’s been surprising to you about the Rapid initiative as you’ve worked on it)?

*PROBES*

- What worked well? Why do you think it worked?
- Unexpected challenges?
- Something that ended up being a waste of time?
- Most effective use of resources?
- What approaches did your team employ in program design, implementation, or operation that you consider innovative or unique?
- Has this initiative led to changes in practice and service delivery? Can you tell me about those changes? (The degree to which things have changed? How significant was the shift for the clinic to offer Rapid on an ongoing basis?)
  - How has RAPID changed your work process (amount or type)?

1. **Sustainability**

*We are curious to learn about factors (context and conditions) that will help and/or hinder sustaining Rapid Start ART services.*

- What do you think Rapid Start ART will look like after the SPNS initiative (and funding) ends?
- Would you say that Rapid Start ART has been integrated into current processes and is standard of care at your organization? How so or how not?
- What support is needed to continue Rapid Start ART services at your organization?
- What funding opportunities (alternative funding sources) and/or resources will your organization need/has your organization identified/secured to continue Rapid Start ART services?
- What changes/modifications/adaptations, if any, do you anticipate having to make to sustain the intervention? Or de-implemented/on hold? [may have been answered previously, select probes as needed]

*PROBES:*

- Changes to staffing?
- Changes to clients served (full sustainment of all RS ART services across all client types, sustainment of RS ART services for newly diagnosed individuals only, or no sustainment of RS ART services)?
- Changes to data monitoring/tracking?
- Changes to community partnerships?
  - Which collaborative partners will be needed to continue with Rapid Start ART?
- For the HRSA/SPNS initiative, each of the sites had to develop a Rapid Start ART protocol. How is that protocol going to be applied/utilized in the future, if at all, at your organization? Will it be modified?

**Staffing**

- Are there any differences in the staff needed to establish and implement Rapid Start ART and the staff needed to sustain Rapid Start ART? Can you talk more about that?

*PROBE:* How do you onboard new staff?

- Is it essential to have someone oversee/in charge of Rapid Start ART to sustain services beyond the SPNS initiative?
  - Who will lead Rapid Start ART services at your organization after the SPNS initiative (and funding) ends?
- Other than the formal Rapid Start ART/implementation leaders, are there people in your organization who will continue to champion/are likely to champion the intervention after the SPNS initiative (and funding) ends?
  - What is the role of the rapid champion at this point in time/moving forward?

**Leadership Engagement**

- In your opinion, what support is needed from leadership to continue Rapid Start ART services at your organization?

*PROBES*

- - What is the current state of leadership involvement with/level of support for Rapid Start ART?
  - Have there been any changes to leadership’s role (level of support/involvement) in this initiative/intervention since implementation?

**Relative Priority - Competing Internal/External Priorities**

- To what extent might Rapid Start ART take a backseat to other high-priority activities or initiatives going on now in your organization?
- How will you manage competing priorities in your own work? How will your colleagues manage these priorities?

1. **Level of Endorsement and Recommendations**

- What advice would you give to sites about steps to take for sustaining activities and shifting away from a “special project” (like this initiative)?
- If you could go back in time and tell yourself one or two things at the beginning of this process, what would it be?
- What lessons would you pass on to other health organizations that want to implement (and sustain) a program like yours?
- Are there any final tips, advice, or words of wisdom you would like to share?
- Do we have permission to circle back with you over email to ask follow-up questions?
- Do you have any questions for us?
- **Would like us to send you a $50 Amazon e-gift card as a Thank you for your time and participation?**
- **If yes, which email address should we send the gift card to?**

Thank you for your time and participation!
